# Mortality of Philadelphia

**Published:** 1858-01

**Authors:** Wilson Jewell


ART. XII.—*Mortality of Philadelphia, for the months of July, August, and September,* 1857; *collated from the Health-Office Record.* By WILSON JEWELL, M.D.

DURING the third quarter there have been 3269 deaths placed on record at the Health-Office. The number of days represented was 98, making 14 weeks, from June 27th to October 3d. This furnishes the average of $33\frac{1}{3}$ deaths per diem.

By reference to the same period in 1856, and which embraced only 13 weeks, or 91 days, it will be seen that the deaths amounted to 3971, or *plus* 702 those of the present quarter, being an equivalent of 9·69 per cent.

Taking the average deaths for the like quarters of 1852, '53, '54, '55, and '56, which would be 3519, it will still leave the deaths for the present quarter 3·68 per cent. below the average.

From these comparisons of the deaths during the third quarter of each of the above years, when we look for an increased mortality as the result of the usual endemics of the season, the conclusion is, that we have passed through a remarkably healthy summer, and have been entirely free from any infectious or epidemic disease.

The deaths charged to certified diseases number 2679, leaving 590, or 18 per cent., as the result of debility, external causes, old age, stillborn, and unknown.

The deaths from stillborn were 167. Of these, 61·08 per cent. were males, and 38·92 per cent. females, or 102 males and 65 females.

Of the sexes, we find 1776 males and 1493 females, showing an excess of male deaths equal to 8·65 per cent.

The mortality among children under one year (exclusive of stillborn) was 1126, or 36·29 per cent. Of those under five years it was 1774, or 57·18 per cent.; while all under twenty years amounted to 2002, or 64·53 per cent. These figures show that the deaths decrease rapidly in proportion to the age from the expiration of one year up to twenty, that those within the first year, exceed those from over one year up to twenty by 12·48 per cent.

During the first decennial period of manhood—that is, between twenty and thirty years—the mortality is greater than at any other, amounting to 274; and from this decenniary onward to extreme old age there is a gradual diminution.

Between the ages of ninety and one hundred there were 14 deaths. The highest number of deaths reached in any one week was 324, between the 22d and 29th of July; of these, 219, or 64 per cent., were children. But in the week ending August 1st, there were 227 children out of 314 deaths. In this week, therefore, 72 per cent. of the mortality was among children.

During this quarter the deaths from the Almshouse made up 5 per cent. of the mortality; while the colored population of the city contributed 4 per cent.

The "Endemic Diseases" furnished a far less proportion of the deaths than is usual for the third quarter of the year, amounting in this instance to 932 only, or 24 per cent. less than those for the preceding year, and falling 10 per cent. below the average of deaths for the third quarters of the past five years.

The most perceptible change will be found in the falling off in the deaths from cholera infantum, scarlet fever, dysentery, and measles. The deaths from fevers of all types amounted only to 131; while last year they contributed 295, a difference of 38 per cent. in favor of the present quarter.

The deaths from cholera infantum are less by 14 per cent. than those for the third quarter of 1856; dysentery, 23 per cent.; scarlet fever, nearly 50 per cent., and measles, 94 per cent.

The deaths from "Sporadic Diseases" have been 7 per cent. less than those for the third quarter of 1856.

From "Nervous Diseases" there were 583 deaths; of these, 406, or 69 per cent., were under five years of age. Convulsions alone furnished 32 per cent., and inflammation of the brain 18 per cent.

The deaths from diseases of the "Organs of Respiration" show a slight increase over those for the like period of 1856. This increase is made up by the deaths from pulmonary consumption and inflammation of the lungs.

As compared with the deaths for the third quarter of 1856, we find that from the "Organs of Circulation," which furnish 70 deaths, there is an increase of 12 per cent. This increased mortality is charged to "Diseases of the Heart," (what diseases?)

Under the head, "Digestive Organs," we notice 98 deaths from "Inflammation of the stomach and bowels,"—double those of last year, which were only 49.

In the deaths from diseases of the "Organs of Generation," which count only 8, there is a decrease of two-thirds from those of last year. The most perceptible improvement is in those from puerperal fever and childbed. Last year they amounted to 17, whereas in the quarter under review they were only 6.

The deaths from "Old Age" have been 61,—an increase of 25 over those of 1856.

The deaths from "External Causes" present only a limited increase.

The "Stillborn," however, have advanced 9 per cent., compared with those for the third quarter of 1856.

### TABLE No. 1.

*Deaths for the Third Quarter of* 1857, *classified.*

| | July. | August. | Sept. | MALE. July | MALE. Aug. | MALE. Sept | FEMALE. July | FEMALE. Aug. | FEMALE. Sept | Total. |
|---|---|---|---|---|---|---|---|---|---|---|
| 1. *Endemic and Contagious Diseases.* Zymotic or Epidemic ............ | 323 | 390 | 219 | 175 | 211 | 132 | 148 | 179 | 87 | 932 |
| 2. *Uncertain or General Seat.* Sporadic Diseases ................ | 153 | 181 | 185 | 81 | 99 | 86 | 72 | 82 | 99 | 519 |
| 3. Nervous System........................ | 226 | 192 | 165 | 130 | 100 | 92 | 96 | 92 | 73 | 583 |
| 4. Organs of Respiration .............. | 202 | 157 | 179 | 106 | 78 | 97 | 96 | 79 | 82 | 538 |
| 5. " Circulation................ | 26 | 29 | 15 | 10 | 17 | 6 | 16 | 12 | 9 | 70 |
| 6. Digestive Organs..................... | 53 | 57 | 48 | 23 | 23 | 23 | 30 | 34 | 25 | 158 |
| 7. Urinary Organs........................ | 4 | 4 | ..... | 3 | 2 | ..... | 1 | 2 | ..... | 8 |
| 8. Organs of Generation............... | 2 | 5 | 1 | ..... | ..... | ..... | 2 | 5 | 1 | 8 |
| 9. " Locomotion.............. | 4 | 5 | 1 | 4 | 4 | 1 | ..... | 1 | ..... | 10 |
| 10. Integumentary System.............. | 3 | 1 | ..... | 2 | ..... | ..... | 1 | 1 | ..... | 4 |
| 11. Old Age ................................ | 19 | 28 | 14 | 5 | 11 | 3 | 14 | 17 | 11 | 61 |
| 12. External Causes ........................ | 51 | 33 | 56 | 42 | 27 | 43 | 9 | 6 | 13 | 140 |
| Stillborn ................................ | 63 | 39 | 65 | 37 | 23 | 42 | 26 | 16 | 23 | 167 |
| Unknown.................................... | 33 | 25 | 13 | 19 | 15 | 4 | 14 | 10 | 9 | 71 |
| | 1162 | 1146 | 961 | 637 | 610 | 529 | 525 | 536 | 432 | 3269 |

### TABLE No. 2.

1. *Endemic and Contagious Diseases—Zymotic or Epidemic.*

| | Males. | Females. | Boys. | Girls. | Under 1 yr. | 1 to 2. | 2 to 5. | 5 to 10. | 10 to 15. | 15 to 20. | 20 to 30. | 30 to 40. | 40 to 50. | 50 to 60. | 60 to 70. | 70 to 80. | 80 to 90. | 90 to 100. | 100 to 110. | Total. |
|---|---|---|---|---|---|---|---|---|---|---|---|---|---|---|---|---|---|---|---|---|
| Cholera.................................... | 1 | ..... | ..... | ..... | ..... | ..... | ... | ... | ... | ... | ... | ... | 1 | ... | ... | ... | ... | ... | ... | 1 |
| " Infantum .......................... | 278 | 230 | 278 | 230 | 336 | 152 | 12 | 5 | 2 | 1 | ... | ... | ... | ... | ... | ... | ... | ... | ... | 508 |
| " Morbus.............................. | 4 | 5 | 4 | 2 | 1 | 2 | 3 | ... | ... | ... | 2 | 1 | ... | ... | ... | ... | ... | ... | ... | 9 |
| Croup ...................................... | 25 | 6 | 25 | 6 | 12 | 9 | 8 | 1 | 1 | ... | ... | ... | ... | ... | ... | ... | ... | ... | ... | 31 |
| Diarrhœa.................................. | 39 | 27 | 36 | 21 | 39 | 15 | 1 | 1 | ... | 1 | 3 | ... | 1 | 2 | 1 | 2 | ... | ... | ... | 66 |
| Dysentery ................................. | 82 | 62 | 53 | 39 | 34 | 32 | 10 | 6 | 5 | 5 | 7 | 10 | 8 | 6 | 14 | 4 | 2 | 1 | ... | 144 |
| Erysipelas ................................ | 8 | 7 | 4 | 4 | 6 | 1 | ... | ... | ... | 1 | 2 | 2 | 1 | 1 | 1 | ... | ... | ... | ... | 15 |
| Fever, Bilious............................ | 6 | 1 | 1 | ..... | ..... | 1 | ... | ... | ... | ... | 1 | 3 | ... | 2 | ... | ... | ... | ... | ... | 7 |
| " Congestive ........................ | 2 | 1 | 1 | 1 | 2 | ..... | ... | ... | ... | ... | ... | ... | 1 | ... | ... | ... | ... | ... | ... | 3 |
| " Gastric .............................. | ..... | 1 | ..... | ..... | ..... | ..... | ... | ... | ... | ... | ... | ... | ... | ... | ... | ... | 1 | ... | ... | 1 |
| " Remittent ......................... | 3 | 1 | 3 | 1 | 1 | 1 | 1 | 1 | ... | ... | ... | ... | ... | ... | ... | ... | ... | ... | ... | 4 |
| " Scarlet.............................. | 26 | 34 | 25 | 34 | 7 | 15 | 23 | 12 | 2 | ... | ... | 1 | ... | ... | ... | ... | ... | ... | ... | 60 |
| " Typhoid ............................ | 20 | 25 | 6 | 10 | ..... | 2 | 3 | 3 | 1 | 7 | 11 | 9 | 4 | 2 | 2 | 1 | ... | ... | ... | 45 |
| " Typhus .............................. | 8 | 3 | 1 | 2 | ..... | ..... | ... | ... | 1 | 2 | 2 | 5 | 1 | ... | ... | ... | ... | ... | ... | 11 |
| Hooping-Cough ......................... | 8 | 5 | 8 | 5 | 8 | 3 | 2 | ... | ... | ... | ... | ... | ... | ... | ... | ... | ... | ... | ... | 13 |
| Influenza .................................. | 4 | 3 | 4 | 3 | 4 | 2 | ... | ... | 1 | ... | ... | ... | ... | ... | ... | ... | ... | ... | ... | 7 |
| Measles .................................... | ..... | 2 | ..... | 2 | ..... | 2 | ... | ... | ... | ... | ... | ... | ... | ... | ... | ... | ... | ... | ... | 2 |
| Smallpox .................................. | 4 | 1 | 4 | 1 | 1 | 1 | 2 | 1 | ... | ... | ... | ... | ... | ... | ... | ... | ... | ... | ... | 5 |
| | 518 | 414 | 453 | 361 | 451 | 238 | 65 | 30 | 13 | 17 | 28 | 31 | 17 | 13 | 18 | 7 | 3 | 1 | ... | 932 |

2. *Uncertain or General Seat—Sporadic Diseases.*

| | Males. | Females. | Boys. | Girls. | Under 1 yr. | 1 to 2. | 2 to 5. | 5 to 10. | 10 to 15. | 15 to 20. | 20 to 30. | 30 to 40. | 40 to 50. | 50 to 60. | 60 to 70. | 70 to 80. | 80 to 90. | 90 to 100. | 100 to 110. | Total. |
|---|---|---|---|---|---|---|---|---|---|---|---|---|---|---|---|---|---|---|---|---|
| Cyanosis | 7 | 1 | 7 | 1 | 4 | 3 | ... | 1 | ... | ... | ... | ... | ... | ... | ... | ... | ... | ... | ... | 8 |
| Abscess | 2 | 7 | ..... | 3 | 1 | 1 | 1 | ... | ... | ... | 2 | ... | 2 | 1 | 1 | ... | ... | ... | ... | 9 |
| Cancer | 9 | 22 | 3 | 2 | ..... | 1 | 3 | ... | ... | 1 | 1 | 5 | 3 | 10 | 6 | 1 | ... | ... | ... | 31 |
| Debility | 67 | 48 | 47 | 31 | 68 | 7 | 1 | 1 | 1 | ... | 7 | 3 | 3 | 9 | 7 | 4 | 4 | ... | ... | 115 |
| Dropsy | 27 | 18 | 5 | 4 | ..... | 2 | 5 | ... | 2 | ... | 4 | 9 | 8 | 5 | 5 | 4 | 1 | ... | ... | 45 |
| Gangrene | 1 | ..... | 1 | ..... | ..... | ..... | ... | 1 | ... | ... | ... | ... | ... | ... | ... | ... | ... | ... | ... | 1 |
| Gout | ..... | 1 | ..... | ..... | ..... | ..... | ... | ... | ... | ... | ... | ... | ... | ... | ... | ... | 1 | ... | ... | 1 |
| Hemorrhage | 3 | 7 | 1 | 2 | 1 | 1 | ... | ... | 1 | ... | 1 | 4 | 1 | ... | ... | 1 | ... | ... | ... | 10 |
| Inflammation | 1 | ..... | 1 | ..... | 1 | ..... | ... | ... | ... | ... | ... | ... | ... | ... | ... | ... | ... | ... | ... | 1 |
| " Throat | ..... | 1 | ..... | 1 | ..... | ..... | 1 | ... | ... | ... | ... | ... | ... | ... | ... | ... | ... | ... | ... | 1 |
| Inanition | 16 | 6 | 13 | 5 | 18 | ..... | ... | ... | ... | ... | ... | 1 | ... | ... | 1 | 1 | ... | 1 | ... | 22 |
| Marasmus | 117 | 114 | 117 | 111 | 162 | 47 | 14 | 4 | 1 | ... | ... | 1 | 2 | ... | ... | ... | ... | ... | ... | 231 |
| Scrofula | 5 | 8 | 3 | 6 | 4 | 3 | ... | ... | 2 | ... | 1 | 3 | ... | ... | ... | ... | ... | ... | ... | 13 |
| Sore Mouth | ..... | 2 | ..... | 2 | 1 | ..... | 1 | ... | ... | ... | ... | ... | ... | ... | ... | ... | ... | ... | ... | 2 |
| Sore Throat | 1 | ..... | 1 | ..... | ..... | 1 | ... | ... | ... | ... | ... | ... | ... | ... | ... | ... | ... | ... | ... | 1 |
| Tabes Messenterica | 10 | 14 | 10 | 14 | 20 | 3 | 1 | ... | ... | ... | ... | ... | ... | ... | ... | ... | ... | ... | ... | 24 |
| Tumor | ..... | 2 | ..... | ..... | ..... | ..... | ... | ... | ... | ... | ... | 1 | ... | ... | 1 | ... | ... | ... | ... | 2 |
| Ulceration | ..... | 2 | ..... | ..... | ..... | ..... | ... | ... | ... | ... | 1 | 1 | ... | ... | ... | ... | ... | ... | ... | 2 |
| | 266 | 253 | 209 | 182 | 280 | 69 | 27 | 7 | 7 | 1 | 17 | 28 | 19 | 25 | 21 | 11 | 6 | 1 | ... | 519 |

3. *Nervous System.*

| | Males. | Females. | Boys. | Girls. | Under 1 yr. | 1 to 2. | 2 to 5. | 5 to 10. | 10 to 15. | 15 to 20. | 20 to 30. | 30 to 40. | 40 to 50. | 50 to 60. | 60 to 70. | 70 to 80. | 80 to 90. | 90 to 100. | 100 to 110. | Total. |
|---|---|---|---|---|---|---|---|---|---|---|---|---|---|---|---|---|---|---|---|---|
| Apoplexy | 20 | 6 | ..... | ..... | ..... | ..... | ... | ... | ... | ... | ... | 4 | 7 | 5 | 6 | 4 | ... | ... | ... | 26 |
| Congestion of Brain | 33 | 27 | 16 | 17 | 21 | 6 | 3 | 2 | ... | 1 | 8 | 4 | 6 | 4 | 3 | 2 | ... | ... | ... | 60 |
| Convulsions | 100 | 88 | 93 | 83 | 115 | 44 | 12 | 5 | ... | ... | 3 | 2 | 1 | 2 | 4 | ... | ... | ... | ... | 188 |
| Coup de Soliel | 6 | ..... | 1 | ..... | ..... | ..... | ... | 1 | ... | ... | 2 | 2 | ... | 1 | ... | ... | ... | ... | ... | 6 |
| Cramp | 3 | 2 | 3 | 2 | 3 | ..... | 1 | ... | 1 | ... | ... | ... | ... | ... | ... | ... | ... | ... | ... | 5 |
| Disease of the Brain | 20 | 17 | 12 | 15 | 20 | 6 | 1 | ... | ... | ... | 2 | 3 | 2 | 2 | 1 | ... | ... | ... | ... | 37 |
| Dropsy of the Brain | 37 | 29 | 37 | 25 | 29 | 23 | 10 | ... | ... | ... | 1 | 1 | ... | ... | 2 | ... | ... | ... | ... | 66 |
| Effusion of the Brain | 16 | 23 | 13 | 21 | 15 | 12 | 4 | 3 | ... | ... | 2 | ... | 1 | ... | 2 | ... | ... | ... | ... | 39 |
| Epilepsy | 1 | ..... | ..... | ..... | ..... | ..... | ... | ... | ... | ... | ... | 1 | ... | ... | ... | ... | ... | ... | ... | 1 |
| Fever of the Brain | ..... | 1 | ..... | 1 | ..... | 1 | ... | ... | ... | ... | ... | ... | ... | ... | ... | ... | ... | ... | ... | 1 |
| Inflammation of the Brain | 55 | 50 | 42 | 42 | 43 | 14 | 19 | 5 | 2 | 1 | 8 | 2 | 6 | 2 | 2 | 1 | ... | ... | ... | 105 |
| Mania-a-Potu | 14 | 3 | ..... | ..... | ..... | ..... | ... | ... | ... | ... | 1 | 5 | 10 | 1 | ... | ... | ... | ... | ... | 17 |
| Palsy | 15 | 14 | 1 | 2 | 1 | ..... | 1 | ... | ... | 1 | 1 | 2 | 2 | 7 | 9 | 4 | 1 | ... | ... | 29 |
| Softening of the Brain | ..... | 1 | ..... | ..... | ..... | ..... | ... | ... | ... | ... | ... | ... | ... | ... | 1 | ... | ... | ... | ... | 1 |
| Tetanus | 2 | ..... | 2 | ..... | 1 | ..... | 1 | ... | ... | ... | ... | ... | ... | ... | ... | ... | ... | ... | ... | 2 |
| | 322 | 261 | 220 | 208 | 248 | 106 | 52 | 16 | 3 | 3 | 28 | 26 | 35 | 24 | 30 | 11 | 1 | ... | ... | 583 |

### 4. *Organs of Respiration.*

| | Males. | Females. | Boys. | Girls. | Under 1 yr. | 1 to 2. | 2 to 5. | 5 to 10. | 10 to 15. | 15 to 20. | 20 to 30. | 30 to 40. | 40 to 50. | 50 to 60. | 60 to 70. | 70 to 80. | 80 to 90. | 90 to 100. | 100 to 110. | Total. |
|---|---|---|---|---|---|---|---|---|---|---|---|---|---|---|---|---|---|---|---|---|
| Asthma | 3 | 4 | 2 | 2 | 4 | ... | ... | ... | ... | ... | ..... | 1 | ... | 2 | ... | ... | ... | ... | ... | 7 |
| Congestion of the Lungs | 17 | 12 | 9 | 6 | 5 | 3 | 3 | 2 | 2 | ... | 3 | 6 | 2 | 1 | ... | 2 | ... | ... | ... | 29 |
| Consumption of the Lungs | 198 | 188 | 48 | 44 | 14 | 16 | 5 | 8 | 8 | 41 | 117 | 86 | 42 | 32 | 12 | 5 | ... | ... | ... | 386 |
| Disease of the Lungs | 3 | 2 | 1 | ... | ... | ... | 1 | ... | ... | ... | 1 | 1 | 1 | ... | 1 | ... | ... | ... | ... | 5 |
| Dropsy of Chest | 5 | 6 | 3 | 2 | 1 | 1 | 1 | ... | 1 | 1 | ..... | ... | 1 | 2 | 1 | 1 | 1 | ... | ... | 11 |
| Hemorrhage of Lungs | 2 | ..... | 1 | ... | 1 | ... | ... | ... | ... | ... | ..... | ... | 1 | ... | ... | ... | ... | ... | ... | 2 |
| Inflammation of Bronchi | 10 | 10 | 7 | 4 | 8 | 1 | 1 | ... | ... | 1 | 1 | 1 | 2 | 1 | 2 | 2 | ... | ... | ... | 20 |
| " Chest | 3 | 3 | 3 | 3 | 3 | 1 | 1 | 1 | ... | ... | ..... | ... | ... | ... | ... | ... | ... | ... | ... | 6 |
| " Larynx | ..... | 1 | ... | 1 | ... | 1 | ... | ... | ... | ... | ..... | ... | ... | ... | ... | ... | ... | ... | ... | 1 |
| " Lungs | 36 | 29 | 21 | 20 | 20 | 10 | 5 | 5 | ... | 1 | 7 | 4 | 6 | 3 | 3 | 1 | ... | ... | ... | 65 |
| " Pleura | 3 | 1 | ... | ... | ... | ... | ... | ... | ... | ... | ..... | ... | 1 | 2 | ... | ... | 1 | ... | ... | 4 |
| " Tonsils | 1 | ..... | 1 | ... | 1 | ... | ... | ... | ... | ... | ..... | ... | ... | ... | ... | ... | ... | ... | ... | 1 |
| " Trachea | ..... | 1 | ... | 1 | 1 | ... | ... | ... | ... | ... | ..... | ... | ... | ... | ... | ... | ... | ... | ... | 1 |
| | 281 | 257 | 96 | 83 | 58 | 33 | 17 | 16 | 11 | 44 | 129 | 99 | 56 | 43 | 19 | 11 | 2 | ... | ... | 538 |

### 5. *Organs of Circulation.*

| | Males. | Females. | Boys. | Girls. | Under 1 yr. | 1 to 2. | 2 to 5. | 5 to 10. | 10 to 15. | 15 to 20. | 20 to 30. | 30 to 40. | 40 to 50. | 50 to 60. | 60 to 70. | 70 to 80. | 80 to 90. | 90 to 100. | 100 to 110. | Total. |
|---|---|---|---|---|---|---|---|---|---|---|---|---|---|---|---|---|---|---|---|---|
| Anæmia | 3 | 2 | 2 | ... | 2 | ... | ... | ... | ... | ... | ... | ... | ... | ... | 1 | 2 | ... | ... | ... | 5 |
| Disease of Heart | 22 | 28 | 5 | 6 | 5 | 2 | ... | ... | 3 | 1 | 5 | 13 | 6 | 3 | 7 | 5 | ... | ... | ... | 50 |
| Dropsy of Heart | 5 | 6 | 1 | 3 | ... | ... | 1 | 1 | 1 | 1 | 1 | ... | 3 | 2 | 1 | ... | ... | ... | ... | 11 |
| Enlargement of Heart | 1 | 1 | ... | ... | ... | ... | ... | ... | ... | ... | ... | 1 | 1 | ... | ... | ... | ... | ... | ... | 2 |
| Gout of Heart | 1 | ... | ... | ... | ... | ... | ... | ... | ... | ... | ... | ... | 1 | ... | ... | ... | ... | ... | ... | 1 |
| Inflammation of Heart | 1 | ... | ... | ... | ... | ... | ... | ... | ... | ... | 1 | ... | ... | ... | ... | ... | ... | ... | ... | 1 |
| | 33 | 37 | 8 | 9 | 7 | 2 | 1 | 1 | 4 | 2 | 7 | 14 | 11 | 5 | 9 | 7 | ... | ... | ... | 70 |

### 6. *Digestive Organs.*

| | Males. | Females. | Boys. | Girls. | Under 1 yr. | 1 to 2. | 2 to 5. | 5 to 10. | 10 to 15. | 15 to 20. | 20 to 30. | 30 to 40. | 40 to 50. | 50 to 60. | 60 to 70. | 70 to 80. | 80 to 90. | 90 to 100. | 100 to 110. | Total. |
|---|---|---|---|---|---|---|---|---|---|---|---|---|---|---|---|---|---|---|---|---|
| Cirrhosis of Liver | 1 | ... | ... | ... | ... | ... | ... | ... | ... | ... | ... | ... | ... | 1 | ... | ... | ... | ... | ... | 1 |
| Colic | ... | 1 | ... | ... | ... | ... | ... | ... | ... | ... | ... | 1 | ... | ... | ... | ... | ... | ... | ... | 1 |
| Consumption of Bowels | 1 | 3 | ... | 2 | 1 | ... | ... | 1 | ... | ... | ... | ... | 1 | ... | 1 | ... | ... | ... | ... | 4 |
| Disease of Liver | 4 | 4 | 2 | ... | 1 | 1 | ... | ... | ... | ... | ... | ... | 2 | 2 | 1 | 1 | ... | ... | ... | 8 |
| Disease of Stomach and Bowels | 1 | 2 | ... | 2 | 2 | ... | ... | ... | ... | ... | ... | 1 | ... | ... | ... | ... | ... | ... | ... | 3 |
| Dropsy of Liver | 1 | ... | ... | ... | ... | ... | ... | ... | ... | ... | ... | ... | 1 | ... | ... | ... | ... | ... | ... | 1 |
| Dropsy of Abdomen | 1 | 3 | ... | ... | ... | ... | ... | ... | ... | ... | ... | 1 | 1 | ... | 1 | 1 | ... | ... | ... | 4 |
| Dyspepsia | 1 | ... | 1 | ... | ... | 1 | ... | ... | ... | ... | ... | ... | ... | ... | ... | ... | ... | ... | ... | 1 |
| Hernia | 1 | 1 | ... | 1 | ... | ... | 1 | ... | ... | ... | ... | 1 | ... | ... | ... | ... | ... | ... | ... | 2 |
| " Strangulated | ... | 1 | ... | ... | ... | ... | ... | ... | ... | ... | ... | ... | ... | ... | 1 | ... | ... | ... | ... | 1 |
| Icterus | ... | 2 | ... | ... | ... | ... | ... | ... | ... | ... | ... | ... | ... | ... | 1 | 1 | ... | ... | ... | 2 |
| Inflammation of Liver | 6 | 4 | 2 | ... | 1 | ... | 1 | ... | ... | ... | ... | 2 | 2 | 3 | 1 | ... | ... | ... | ... | 10 |
| " Peritoneum | 2 | 8 | 1 | 3 | ... | 1 | ... | ... | 1 | 2 | ... | 3 | 1 | 2 | ... | ... | ... | ... | ... | 10 |
| " Stomach and Bowels | 43 | 55 | 26 | 29 | 31 | 9 | 8 | 3 | ... | 4 | 10 | 11 | 8 | 6 | 5 | 3 | ... | ... | ... | 98 |
| Obstruction of the Bowels | ... | 1 | ... | ... | ... | ... | ... | ... | ... | ... | ... | ... | ... | ... | 1 | ... | ... | ... | ... | 1 |
| Scirrhus of Intestines | 1 | ... | ... | ... | ... | ... | ... | ... | ... | ... | ... | 1 | ... | ... | ... | ... | ... | ... | ... | 1 |
| Teething | 3 | 3 | 3 | 3 | 4 | 2 | ... | ... | ... | ... | ... | ... | ... | ... | ... | ... | ... | ... | ... | 6 |
| Ulceration of Bowels | 1 | 1 | ... | 1 | ... | ... | ... | 1 | ... | ... | ... | 1 | ... | ... | ... | ... | ... | ... | ... | 2 |
| Worms | 2 | ... | 2 | ... | 1 | ... | ... | 1 | ... | ... | ... | ... | ... | ... | ... | ... | ... | ... | ... | 2 |
| | 69 | 89 | 36 | 41 | 41 | 14 | 10 | 6 | 1 | 6 | 10 | 22 | 16 | 14 | 12 | 6 | ... | ... | ... | 158 |

7. *Urinary Organs.*

| | Males. | Females. | Boys. | Girls. | Under 1 yr. | 1 to 2. | 2 to 5. | 5 to 10. | 10 to 15. | 15 to 20. | 20 to 30. | 30 to 40. | 40 to 50. | 50 to 60. | 60 to 70. | 70 to 80. | 80 to 90. | 90 to 100. | 100 to 110. | Total. |
|---|---|---|---|---|---|---|---|---|---|---|---|---|---|---|---|---|---|---|---|---|
| Albuminuria | 1 | 1 | ... | ... | ... | ... | ... | ... | ... | ... | 2 | ... | ... | ... | ... | ... | ... | ... | ... | 2 |
| Disease of Kidneys | 1 | 1 | 1 | ... | ... | ... | ... | 1 | ... | ... | ... | ... | ... | ... | 1 | ... | ... | ... | ... | 2 |
| Inflammation of Bladder | 2 | 1 | ... | ... | ... | ... | ... | ... | ... | ... | ... | ... | ... | 1 | 2 | ... | ... | ... | ... | 3 |
| " Kidneys | 1 | ... | ... | ... | ... | ... | ... | ... | ... | ... | 1 | ... | ... | ... | ... | ... | ... | ... | ... | 1 |
| | 5 | 3 | 1 | ... | ... | ... | ... | 1 | ... | ... | 3 | ... | ... | 1 | 3 | ... | ... | ... | ... | 8 |

8. *Organs of Generation.*

| | Males. | Females. | Boys. | Girls. | Under 1 yr. | 1 to 2. | 2 to 5. | 5 to 10. | 10 to 15. | 15 to 20. | 20 to 30. | 30 to 40. | 40 to 50. | 50 to 60. | 60 to 70. | 70 to 80. | 80 to 90. | 90 to 100. | 100 to 110. | Total. |
|---|---|---|---|---|---|---|---|---|---|---|---|---|---|---|---|---|---|---|---|---|
| Childbed | ... | 3 | ... | ... | ... | ... | ... | ... | ... | ... | 2 | 1 | ... | ... | ... | ... | ... | ... | ... | 3 |
| Fever, Puerperal | ... | 3 | ... | ... | ... | ... | ... | ... | ... | ... | 2 | 1 | ... | ... | ... | ... | ... | ... | ... | 3 |
| Hemorrhage from Uterus | ... | 1 | ... | ... | ... | ... | ... | ... | ... | ... | 1 | ... | ... | ... | ... | ... | ... | ... | ... | 1 |
| Inflammation of Uterus | ... | 1 | ... | 1 | ... | ... | ... | ... | ... | 1 | ... | ... | ... | ... | ... | ... | ... | ... | ... | 1 |
| | ... | 8 | ... | 1 | ... | ... | ... | ... | ... | 1 | 5 | 2 | ... | ... | ... | ... | ... | ... | ... | 8 |

9. *Organs of Locomotion.*

| | Males. | Females. | Boys. | Girls. | Under 1 yr. | 1 to 2. | 2 to 5. | 5 to 10. | 10 to 15. | 15 to 20. | 20 to 30. | 30 to 40. | 40 to 50. | 50 to 60. | 60 to 70. | 70 to 80. | 80 to 90. | 90 to 100. | 100 to 110. | Total. |
|---|---|---|---|---|---|---|---|---|---|---|---|---|---|---|---|---|---|---|---|---|
| Coxalgia | 1 | ... | 1 | ... | ... | ... | ... | ... | 1 | ... | ... | ... | ... | ... | ... | ... | ... | ... | ... | 1 |
| Disease of Spine | 3 | 1 | 3 | 1 | 2 | 1 | ... | ... | ... | 1 | ... | ... | ... | ... | ... | ... | ... | ... | ... | 4 |
| Malformation of Spine | 1 | ... | 1 | ... | 1 | ... | ... | ... | ... | ... | ... | ... | ... | ... | ... | ... | ... | ... | ... | 1 |
| Rheumatism | 4 | ... | 1 | ... | ... | ... | ... | ... | 1 | ... | 1 | ... | 1 | 1 | ... | ... | ... | ... | ... | 4 |
| | 9 | 1 | 6 | 1 | 3 | 1 | ... | ... | 2 | 1 | 1 | ... | 1 | 1 | ... | ... | ... | ... | ... | 10 |

10. *Integumentary System.*

| | Males. | Females. | Boys. | Girls. | Under 1 yr. | 1 to 2. | 2 to 5. | 5 to 10. | 10 to 15. | 15 to 20. | 20 to 30. | 30 to 40. | 40 to 50. | 50 to 60. | 60 to 70. | 70 to 80. | 80 to 90. | 90 to 100. | 100 to 110. | Total. |
|---|---|---|---|---|---|---|---|---|---|---|---|---|---|---|---|---|---|---|---|---|
| Carbuncle | ... | 1 | ... | ... | ... | ... | ... | ... | ... | ... | ... | ... | 1 | ... | ... | ... | ... | ... | ... | 1 |
| Eruptions | 1 | ... | 1 | ... | ... | 1 | ... | ... | ... | ... | ... | ... | ... | ... | ... | ... | ... | ... | ... | 1 |
| Ozena Maligna | ... | 1 | ... | ... | ... | ... | ... | ... | ... | ... | ... | 1 | ... | ... | ... | ... | ... | ... | ... | 1 |
| Ulceration of Scalp | 1 | ... | ... | ... | ... | ... | ... | ... | ... | ... | ... | ... | ... | ... | ... | 1 | ... | ... | ... | 1 |
| | 2 | 2 | 1 | ... | ... | 1 | ... | ... | ... | ... | ... | 1 | 1 | ... | ... | 1 | ... | ... | ... | 4 |

11. *Old Age.*

| | Males. | Females. | Boys. | Girls. | Under 1 yr. | 1 to 2. | 2 to 5. | 5 to 10. | 10 to 15. | 15 to 20. | 20 to 30. | 30 to 40. | 40 to 50. | 50 to 60. | 60 to 70. | 70 to 80. | 80 to 90. | 90 to 100. | 100 to 110. | Total. |
|---|---|---|---|---|---|---|---|---|---|---|---|---|---|---|---|---|---|---|---|---|
| Old Age | 19 | 42 | ... | ... | ... | ... | ... | ... | ... | ... | ... | ... | ... | 2 | 3 | 15 | 29 | 12 | ... | 61 |

12. *External Causes.*

| | Males. | Females. | Boys. | Girls. | Under 1 yr. | 1 to 2. | 2 to 5. | 5 to 10. | 10 to 15. | 15 to 20. | 20 to 30. | 30 to 40. | 40 to 50. | 50 to 60. | 60 to 70. | 70 to 80. | 80 to 90. | 90 to 100. | 100 to 110. | Total. |
|---|---|---|---|---|---|---|---|---|---|---|---|---|---|---|---|---|---|---|---|---|
| Asphyxia | 2 | 1 | 2 | 1 | 3 | ... | ... | ... | ... | ... | ... | ... | ... | ... | ... | ... | ... | ... | ... | 3 |
| Burns | 1 | 4 | 1 | 4 | 1 | 1 | 1 | ... | ... | 2 | ... | ... | ... | ... | ... | ... | ... | ... | ... | 5 |
| Casualties | 27 | 8 | 8 | 2 | 3 | ... | 2 | 1 | 1 | 3 | 6 | 8 | 3 | 3 | 2 | 2 | 1 | ... | ... | 35 |
| Drowned | 62 | 8 | 21 | 6 | 4 | 1 | 1 | 9 | 5 | 7 | 20 | 12 | 10 | ... | 1 | ... | ... | ... | ... | 70 |
| Fracture | 4 | ... | 1 | ... | 1 | ... | ... | ... | ... | ... | 2 | 1 | ... | ... | ... | ... | ... | ... | ... | 4 |
| " Leg | 1 | ... | ..... | ... | ..... | ... | ... | ... | ... | ... | ... | ... | ... | ... | 1 | ... | ... | ... | ... | 1 |
| " Skull | 2 | ... | 1 | ... | ..... | ... | ... | ... | 1 | ... | 1 | ... | ... | ... | ... | ... | ... | ... | ... | 2 |
| Intemperance | 3 | 4 | ..... | ... | ..... | ... | ... | ... | ... | ... | 1 | 3 | 2 | 1 | ... | ... | ... | ... | ... | 7 |
| Suffocation | ..... | 1 | ..... | 1 | 1 | ... | ... | ... | ... | ... | ... | ... | ... | ... | ... | ... | ... | ... | ... | 1 |
| Suicide | 8 | 2 | ..... | ... | ..... | ... | ... | ... | ... | ... | 2 | 3 | 2 | 3 | ... | ... | ... | ... | ... | 10 |
| Violence | 1 | ... | ..... | ... | ..... | ... | ... | ... | ... | ... | 1 | ... | ... | ... | ... | ... | ... | ... | ... | 1 |
| Wounds | 1 | ... | 1 | ... | ..... | ... | ... | ... | ... | 1 | ... | ... | ... | ... | ... | ... | ... | ... | ... | 1 |
| | 140 | 28 | 35 | 14 | 13 | 2 | 4 | 10 | 7 | 13 | 33 | 27 | 17 | 7 | 4 | 2 | 1 | ... | ... | 140 |
| Stillborn | 102 | 65 | 102 | 65 | 167 | ... | ... | ... | ... | ... | ... | ... | ... | ... | ... | ... | ... | ... | ... | 167 |
| Unknown | 38 | 33 | 19 | 17 | 25 | 5 | 1 | 2 | ... | 3 | 13 | 7 | 9 | 2 | 4 | ... | ... | ... | ... | 71 |

### TABLE No. 3.

*Deaths for the Third Quarter of* 1857, *at fifteen distinct periods of life.*

| | | | |
|---|---|---|---|
| Under 1 year | 1126 | 60 to 70 | 123 |
| 1 to 2 | 471 | 70 to 80 | 71 |
| 2 to 5 | 177 | 80 to 90 | 42 |
| 5 to 10 | 89 | 90 to 100 | 14 |
| 10 to 15 | 48 | 100 to 110 | ..... |
| 15 to 20 | 91 | | |
| 20 to 30 | 274 | | 3102 |
| 30 to 40 | 257 | Stillborn | 167 |
| 40 to 50 | 182 | | |
| 50 to 60 | 137 | Total | 3269 |

Included in the above table are 177 from the Blockley Almshouse; 150 colored people and 27 from the country, to wit:—

| | July. | August. | September. | Total. |
|---|---|---|---|---|
| Almshouse | 57 | 76 | 44 | 177 |
| Colored People | 51 | 62 | 37 | 150 |
| Country | 8 | 8 | 11 | 27 |
| | 116 | 146 | 92 | 354 |

TABLE No. 4.

The following table gives the total number of deaths for each week during the quarter, with the sexes, adults and children:—

| 1857. | Males. | Females. | Adults. | Children. | Total. | Total Monthly. |
|---|---|---|---|---|---|---|
| From June 27th to July 4th | 101 | 73 | 65 | 109 | 174 | ...... |
| " July 4th to July 11th | 110 | 104 | 86 | 128 | 214 | ...... |
| " July 11th to July 18th | 111 | 89 | 78 | 122 | 200 | ...... |
| " July 18th to July 25th | 146 | 114 | 70 | 190 | 260 | ...... |
| " July 25th to August 1st | 170 | 144 | 87 | 227 | 314 | 1162 |
| " August 1st to August 8th | 157 | 144 | 87 | 214 | 301 | ...... |
| " August 8th to August 15th | 148 | 136 | 85 | 199 | 284 | ...... |
| " August 15th to August 22d | 129 | 108 | 87 | 150 | 237 | ...... |
| " August 22d to August 29th | 176 | 148 | 105 | 219 | 324 | 1146 |
| " August 29th to September 5th | 129 | 110 | 69 | 170 | 239 | ...... |
| " September 5th to September 12th | 113 | 102 | 75 | 140 | 215 | ...... |
| " September 12th to September 19th | 111 | 67 | 66 | 112 | 178 | ...... |
| " September 19th to September 26th | 103 | 79 | 83 | 99 | 182 | ...... |
| " September 26th to October 3d | 73 | 74 | 57 | 90 | 147 | 961 |
| Total | 1777 | 1492 | 1100 | 2169 | 3269 | 3269 |

